# Dogs outperform cats both in their testability and relying on human pointing gestures: a comparative study

**DOI:** 10.1038/s41598-023-45008-3

**Published:** 2023-10-19

**Authors:** Attila Salamon, Stefania Uccheddu, Melitta Csepregi, Ádám Miklósi, Márta Gácsi

**Affiliations:** 1 ELKH-ELTE Comparative Ethology Research Group, Budapest, Hungary; 2 MTA-ELTE Comparative Ethology Research Group, Budapest, Hungary; 3 Department of Ethology, Eötvös Loránd University, Budapest, Hungary; 4 Doctoral School of Biology, Institute of Biology, Eötvös Loránd University, Budapest, Hungary

**Keywords:** Neuroscience, Zoology

## Abstract

The dog is a so far unique species to study interspecific communication and a promising evolutionary model for preverbal human communication. Recently cats were reported to show some similar skills to dogs. Here we directly compared both the testability and the success of companion dogs and cats in relying on human distal pointing gestures. Due to differences in their domestication, social and ecological background, and developmental processes, we expected better performance from dogs compared to cats. Using an object-choice task, cats made considerably fewer choices than dogs in the laboratory environment, and their tendency to make a choice declined during trials. They were slightly more testable at home, where their willingness to choose did not decrease over time. Dogs made more successful choices than cats, both at the group and individual level, irrespective of the type of the pointing gesture. Older cats were more successful. Despite the two species’ rather similar role nowadays as human companions, our results support previous findings suggesting that, compared to the dog, the cat is a less ideal model to study some human communicative abilities in a laboratory environment.

## Introduction

Due to its unique domestication history and special role in the human environment, the dog is considered as an important model species for investigating some aspects of the early evolution of human social skills (e.g.,^1,2^) and communicative abilities (e.g.,^3–5^). In recent years, cats also gained popularity in this line of research investigating the effect of domestication on companion animals’ communication skills (e.g.,^5–7^). In comparative studies to investigate different species’ communicative skills and also to gain insight into the evolution of our own species’ comprehension of subtle pointing gestures, the two-way object choice task is a widely used paradigm (e.g.,^8–10^). However, to validly compare the abilities or behaviour of different species, testing in a laboratory environment, where a standard protocol can be followed and most potentially influencing factors can be controlled for, is essential (e.g.,^11–14^).

In the course of their domestication, dogs (*Canis familiaris*) adapted to the new interspecific social environment and developed a set of human-compatible socio-cognitive skills, which may have enhanced their survival in the anthropogenic niche^2,15^. Dogs are sensitive to the attentional state of humans^16,17^, they are able to rely on various forms of human body gestures, such as head turning, bowing, body orientation, pointing with finger, as communicative cues^3,18^. Both puppies^10^ and adult dogs rely on distal pointing gestures (e.g., dynamic-sustained, momentary)^1,19–21^, and adult dogs also seem to comprehend pointing signals, which can be interpreted as referential communicative gestures^22,23^. Dogs’ behaviour in some two-way choice tests even reflected a special sensitivity to human communicative cues and indicated how susceptibility to such human social signals could (mis)lead them^24^.

For thousands of years, cats (*Felis catus*) lived solitarily, close to human settlements^25^ contrary to dogs, not in almost complete dependence^26,27^ and only in the last few hundred years did they become companion animals living with humans^25^. The ancestor of dogs lived in close family groups and had complex social behavior^28^, while the ancestor of the cat was a strongly territorial, solitary animal^29^ with minimal contact between individuals, except during the reproductive period^30^. Territoriality plays an important role in the case of both species, but while cats’ home range, in which they roam comfortably, is fixed and located around a food resource^31^, wolves and feral dogs may have shifts in their home range use to access a food resource that is outside their core territory^32^.

Nowadays, in modern societies both cats and dogs have similar roles as pets in humans’ lives, but there are also some important differences between the two species. Cats are kept as either indoor-only pets or as indoor-outdoor pets with some degree of outdoor access (e.g.,^33,34^), while dogs can be kept indoors, outdoors or both (e.g.,^34,35^). Further, while cats are generally unattended in their outdoor activities, dogs may participate in some form of activity with their owner when outdoors (e.g., dog-walking^36^ or sports^37^). Compared to dogs, the adaptation of cats to the human anthropogenic niche seems less evident, thus, based on their ancestors’ ecology and behaviour, cats are less expected to show similarly developed interspecific socio-cognitive skills than dogs. Still, nowadays cats are highly successful in modern societies as companion animals (in the EU 25% of households have at least one cat, while that number for Hungary is 34%, see^38^). In line with the latter statement, cats were reported to recognize their owners^39^, discriminate some human emotional expressions^40^, while their attachment towards their owners is debated^41^. Cats seem to be able to follow human gazing in ostensive and non-ostensive situations in a two-way object choice task^42^, however, so far, only two studies have directly compared the responses of cats and dogs to human pointing gestures in the subjects’ homes^20,43^. Miklósi et al.^20^ found that at the group level both cats and dogs performed above chance level in four types of pointing gestures (proximal and distal, dynamic and momentary pointing in all combinations). Cats performed better in proximal dynamic pointing than in both proximal and distal momentary pointing, while no such differences were found in dogs. Further, cats and dogs did not differ in their performance in either of the pointing types. Kraus et al.^43^ compared cats’ and dogs’ performance in relying on human pointing cues (proximal pointing with ipsilateral arm + head gaze). At the group level, both dogs and cats performed better than chance, and the performance of the two species did not differ in the peripheral setup.

Since in comparative studies dogs (as well as apes or children) are usually tested in the laboratory environment, one can argue that cats would also need to be tested under the same conditions to yield fully comparable performance. However, cats are reported to be sensitive to a changing, unpredictable and uncontrollable environment, which causes stress to them^30,44,45^. Based on this (and maybe on pilot tests), and without direct comparisons in experimental situations, researchers seemed to presume that testing companion cats in their familiar environment would solve this problem (e.g.,^20,42,46,47^). Most importantly, to be able to test cats in a laboratory (or sometimes even at home), cats are often pre-selected according to their willingness to accept the presence of an unfamiliar person and cooperate with him or her. Most studies testing cats at their homes used pre-selection criteria (e.g., the subject had to approach experimenter within 1 min after being called by its name; subject could be petted by the experimenter for 1 min; and subject ate a piece of food from the bowl offered by the experimenter) from which at least two had to be met to participate in the experiments^20,42,48^. In the study of Merola et al.^49^ a questionnaire was sent to owners in advance to select cats that were used to traveling, are friendly with strangers, and do not show escaping or aggressive behaviours and only these selected cats were tested in the laboratory. Of note, even if selection criteria were used during the recruitment, further subjects were dropped out for various reasons before and/or during the testing (e.g., low motivation, ‘being present but not participating’^20,42,48^). Even in studies carried out at home, the number of cats strongly decreased due to exclusions from their initial enrolment through pre-testing (familiarization and motivation tests) to test completion. In these studies, where cats’ reliance on human social cues and/or ostensive signals was tested, only 41.4–60.9% of cats completed the tests (see Table 1). In contrast, the majority of dogs tend to complete such tests (Table 1), in most papers there is even no report on dropout rate.

**Table 1:** Number of subjects included in previous studies regarding cats’ and dogs’ use of pointing gestures. The authors did not find any studies in which cats were tested in a laboratory setting. Only papers that reported dropout rates at some stage of the experiments are listed here.

Study	Subjects enrolled (N)	Subjects excluded before test (%)	Subjects excluded during test (%)	Subjects completed test (%)	Test location
Cats					
Miklósi et al. (2005), Exp 1	23	8.7	30.4	60.9	Home
Kraus et al. (2014)	30	56.7^a^		43.3	Indoor enclosure^b^
Pongrácz et al. (2019)	99	14.1	44.5	41.4	Home
Pongrácz and Onofer (2020)	68	13.2	30.9	55.9	Home
Mäses and Wascher (2023)	20	50	–	50	Shelter
Dogs					
Miklósi et al. (2005), Exp 1	19	–	–	100^c^	Home
Kraus et al. (2014)	40	–	–	100	Home
Riedel et al. (2008)^d^	150	1.3	2.7	96	Home or lab^e^
Gácsi et al. (2009a)^d^	23	4.3	–	95.7	Lab
Pongrácz et al. (2013)^d^	173	4	2.9	93.1	Lab or outdoors

^a^Kraus et al. (2014) reported the number of cats dropping out during the training and early in the experiment together.

^b^The cats were tested in their indoor enclosure or the familiar hallway in front of their enclosure.

^c^In Miklósi et al. (2005) all dogs completed the testing but 5 were excluded, as the cats living with these dogs dropped out during testing.

^d^In these studies the subjects of all experiments were combined.

^e^Puppies up to 8 weeks old were tested at home, while older puppies and adults were tested in a laboratory.

In the present study our main aims were twofold. We wanted to directly compare both the testability (the number of choices the subjects made) and the performance (success in relying on the human gestures) of the two species, so that we could evaluate their suitability as model species for investigating this element of non-verbal human communication.

We assessed cats’ testability both in the laboratory and at home; dogs were tested only at the laboratory, because we knew from previous studies that their testability is very high (see Table 1). We predicted that even after the habituation process (see^50^), fewer cats than dogs could be successfully tested in the laboratory, and that cats would be more testable in their home environment compared to the laboratory due to their sensitivity to changing environments (e.g.,^30,44,45^). Since Uccheddu et al.^50^ reported that younger cats were easier to habituate to an unfamiliar environment/experimenter, we expected that younger cats would be more testable.

Second, we compared the success of dogs and cats in relying on two types of human given cue, distal dynamic-sustained (DDS) pointing (the signal is on during the subject’s response) and distal momentary (DM) pointing (the signal er keeps the arm in position only for a second, then the arm is taken back to the chest after which the dog can choose), in the laboratory environment. In both two-way object choice tasks, several elements of communication are needed to be applied for success; paying attention to an unfamiliar person (experimenter), inhibition of the subjects’ own actions^10^, recognition of the experimenter’s intention to communicate from her eye-contact and other attention getting signals^51^, comprehending and relying on the human pointing gestures either by recognizing it as an imperative directive ordering of where to go^15,51^ or considering the person pointing trustworthy/reliable^52,53^.

During the domestication of dogs, selection acted in the direction of cooperation with humans and the development of subtle human-related communicative skills^2^. However, this selective pressure was not present in cat domestication as cats did not evolve in close interaction with humans^25^. Thus, we expected that dogs would be more successful than cats, and even if cats relied on the distal pointing gestures^20^, dogs would perform better because, contrary to previous studies, we ensured that the distance between the index finger and the baited bowl appeared to be about 80 cm (see Supplementary Information). Depending on the type of the pointing gesture, it can be interpreted as a referential communicative act, that is, referring to a specific object or location in space (e.g., children^8^) or as a discriminative cue (referring to the side where the hand is moving/kept) (e.g., chimpanzees, *Pan troglodytes*^8^). We expected that in our set up, it would be less probable that the pointing gesture can be interpreted in a non-communicative context (as if the person was trying to reach/touch the baited bowl).

As an alternative hypothesis, it could be assumed that, in contrast to dogs, cats, like wolves^10^, learn to pay attention to and rely on these human gestures during their early development in the human environment, thus as adults they perform similarly to dogs^42^. Based on this developmental hypothesis, we would expect older cats to be more successful in the pointing task, due to their accumulated experience living in the human environment. We noted, however, that Pongrácz et al.^42^ results do not support a learning mechanism, since they found a trend-like association with age in the opposite direction (younger cats seemed to be more successful in a pointing + gazing task).

Finally, we aimed to retest both cats and dogs in the same laboratory location to check if their success increases by experience in the test situation. We did not expect any learning effect in dogs (see^21,54^), but expected some improvement in cats due to habituation to the test situation. Although no such data have been reported on cats so far, we expected cats’ performance to be better at home because of the familiar environment. As the home test was always conducted after the laboratory test, their retest in the laboratory also served as a control for a potential order effect related to the comparison of their laboratory vs. home performance.

## Materials and methods

### Ethical statement

**Figure 1:**
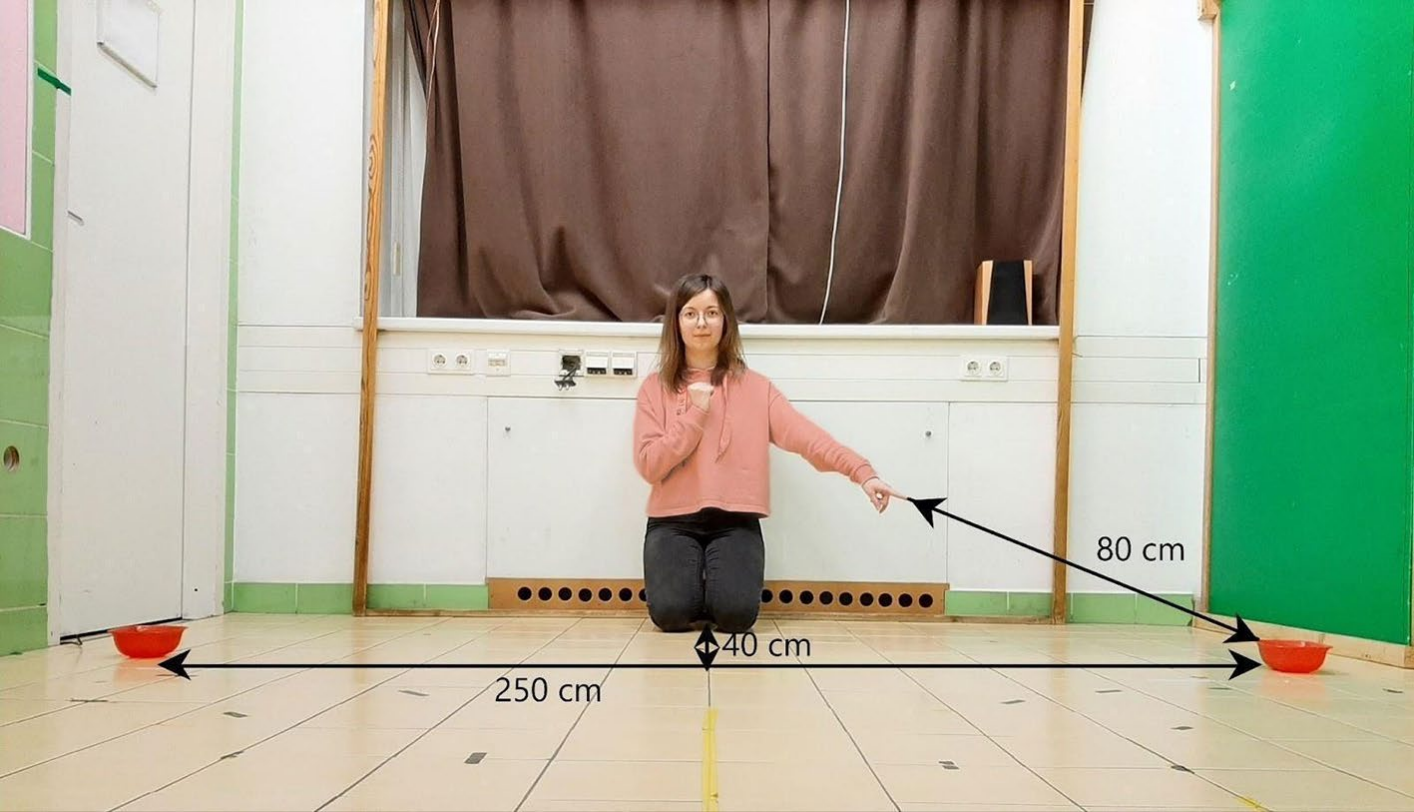
Experimental setup, beginning of a test trial. When the subject pays attention to the attention-getting cue (e.g. clapping or tapping), the experimenter presents a pointing gesture. In the case of DDS pointing the arm stayed in position until the subject chose, while in DM pointing the arm was extended towards one of the containers for a second and then pulled back to the chest, after which the subject was allowed to choose.

All procedures were approved by the Ethical Committee of Eötvös Loránd University (Permission # PE/EA/1005-5/2018). All methods were carried out in accordance with relevant guidelines and regulations, the experiment was performed in accordance with the EU Directive 2010/63/EU and the recommendations of the Hungarian State Health and Medical Service. Owners were recruited via the Family Dog Project and social media. Informed consent was obtained from all owners and they participated in the test with their pets on a voluntary basis. Owners were present at the tests and they were told that they could terminate the experiment at any time when they thought their pet was being exposed to unwanted stress. Informed consents were also obtained from the experimenters visible on Fig. 1 and Supplementary Figure S1 to publish their images in an open-access publication.

### Test location

Since this is a comparative study all information applies to both cats and dogs, unless stated otherwise.

Even though we expected problems with cats as they are sensitive to changing environments^44,45^, we wanted to test our subjects first in the laboratory in order to have a controlled environment and comparable results to that of the dogs’. As companion dogs are typically tested in a laboratory environment according to the established protocols of several leading behavioral research groups^12,55,56^, we planned a home test only for cats, as we expected them to perform better at home due to the calming, familiar environment. A retest in the laboratory was planned for both species as a control for learning effects, however, the cats' laboratory retest had to be cancelled due to the COVID-19 outbreak. The retest was important because so far only the within-test learning effect had been examined (see e.g. ^20,54^) and not the effect of repeated testing.

The laboratory experiments were carried out in a 3 × 5.4 m room of the Department of Ethology, Eötvös Loránd University in the presence of the animal’s owner (O) and a female experimenter (E). In the laboratory, all air-circulation was turned off and the windows were closed during the tests. The floor was washed before each test.

Cats’ home test was always performed after the laboratory test (140 ± 113 days (mean ± SD) later) by the same E as in the laboratory. The test procedure, which is described below, was the same in both locations. At home, the test was done in an approximately 3 × 3 m area (generally a living room), which did not overlap with the cat’s feeding area. If anyone else was at home, they were asked not to eat, cook or make noise (e.g. watch TV) during the test. Any other pets in the household were closed into a separate room for the duration of the test.

#### Habituation

Uccheddu et al.^50^ suggested that, in contrast to dogs, cats require a habituation test/process in order to be eligible for testing in a laboratory environment. In their study, companion cats and dogs participated in a habituation process to be familiarized with the laboratory environment. The subjects were considered as being habituated if they explored the environment and either accepted food from an unfamiliar female E or played with her (see in detail in^50^). As a maximum, three occasions were offered for habituating the subject and to pass the criteria. All dogs but only 40.3% of the cats passed the habituation test on the first occasion. Ultimately 59.7% of the enrolled cats passed during the potential three occasions^50^.

### Subjects

For the current study, we only included subjects that, on a separate occasion, already went through the above mentioned habituation process and fulfilled the criteria based on the method in Uccheddu et al.^50^. Also, the owners were instructed not to feed their cats in the 3 h preceding the test.

Our subjects were tested in the pointing test by the same female E who had performed the habituation test (cats: 2.64 ± 2.45 months (mean ± SD), dogs: 3.75 ± 3.43 months (mean ± SD) after habituation).

Since we expected some cats to drop out during the experiment for various reasons, we aimed to collect about twice as many cats as dogs to ensure that enough cats remained for the analysis.

In total 43 companion cats arrived at the Department of Ethology for the pointing test, but 9 of them were excluded for various reasons (2 did not come out of the box, 5 failed the motivation test, 1 developed a breathing problem and 1 bit the O during the test) and the data of one tested cat could not be used because of the failure of the video recording system (Table 2). Finally, we analyzed the data of 33 cats (mean age = 30.6 ± 30.3 (SD) months, range: 2–138 months; most cats did not belong to any breed with the exception of 1 Siamese and 1 Maine Coon; 17 males (13 neutered), 16 females (10 neutered)).

**Table 2:** The number (and %) of subjects remaining at the beginning of each stage of the study. Both cats and dogs were first tested in the laboratory. Then cats had a test at home. Finally, dogs were retested in the laboratory and a retest was also planned for cats in the laboratory, but it was cancelled due to COVID-19.

Study stages	Analyses	Lab		Home	Lab (Retest)	
		Cats	Dogs	Cats	Dogs	Cats*
Familiarization phase		43	21	23	15	–
Motivation test		41 (95.3%)	21 (100%)	23 (100%)	15 (100%)	–
Pointing test		36 (83.7%)	21 (100%)	21 (100%)	15 (100%)	–
	Choice analysis	33 (76.7%)	21 (100%)	19 (82.6%)	15 (100%)	–
	Success analysis (of subjects that made a choice in at least half of the trials)	15 (34.9%)	21 (100%)	14 (60.9%)	15 (100%)	–

*Canceled due to COVID-19.

All cats lived with the owner for at least 2 months before the test. About two third of the cats (N = 28) were indoor cats. None of the cats have participated in any cognitive test previously.

Out of the 43 cats, due to the COVID-19 lockdown situation, we could only visit a subsample (N = 23) at home. During the home testing, which was always conducted after the laboratory test, we had to exclude 4 cats (2 failed the motivation test, 2 hid behind the bed during the test), so we could use the data of 19 cats (Table 2). Out of these, 5 cats did not have a valid laboratory test result (3 of them failed the motivation test, 1 did not leave the box and 1 was excluded due to technical problems). Thus, we could directly compare the results from the two test locations in the case of 14 cats (mean age ± SD = 36.9 ± 38.5 months; none of the cats belonged to any breed; 7 males (all neutered) and 7 females (4 neutered)).

We tested 21 small sized dogs (max. 30 cm height at withers) in the laboratory (mean age = 52 ± 43.8 (SD) months, range: 6–149 months; 9 males (4 neutered), 12 females (8 neutered). Small dogs were used so that we could enact the pointing gesture the very same way as in the case of cats. We included various breeds to have a sample as representative as possible: 3 Yorkshire terriers, 3 Jack Russell terriers, 2 Maltese, 1 beagle, 1 French bulldog, 1 Havanese, 1 miniature pinscher, 1 papillon, 1 poodle, 1 dachshund, 1 wire-haired dachshund, 1 miniature dachshund, and 4 mongrels. None of the dogs had to be excluded from the pointing tests (Table 2).

### Familiarization phase

Before starting the experiment, for 5 min the subject could freely explore the laboratory, which was the same as the location of the habituation test. Both the E and the O were present; E explained the procedure to the O.

### Motivation test

After the familiarization phase, 4–6 training trials were done (lasting approx. 2–3 min) to ensure that the animals were motivated enough to go for the food reward. For cats, every O brought their cats’ favourite food (small bits up to 4–5 mm in diameter). In the case of the dogs, we used the same hypoallergenic dog treat (1 cm in diameter) for each subject. The E used two identical, red coloured, opaque plastic containers. During the motivation trials the O sat down at a predetermined point restraining the subject by its collar/harness or holding its body. One plastic container was placed right in front of them and the E was kneeling, facing the subject at a position approx. 20 cm behind the container. Immediately after kneeling down the E placed the food in the container in a fully visible way for the subject, and then the O released the subject that could approach and eat the food. This was repeated twice (once with each container), and the subject failed the motivation test, if it did not eat the food. After these, one plastic container was placed 2.5 m away from the subject and the E was kneeling down facing the subject at a position approx. 30–40 cm behind the container. Again, E placed the food in the container in full view of the subject, and then the O released the subject that could approach and eat the food. This was also repeated minimum twice (once with each container), and a maximum of four times. Those subjects that were not motivated to eat the food twice in a row within these 4 trials were also excluded and did not participate in the test trials (N = 5 cats in the laboratory, N = 2 cats at home).

### Pointing test

The pointing test trials immediately followed the motivation trials. For each subject, the same E carried out the familiarization, motivation, and pointing tests. In the test trials the animals were presented with a two-way object choice situation, where the E indicated one of the objects (bowl with hidden food reward) by a pointing gesture in 28 trials with two different test conditions. The two conditions were DDS and DM pointing gestures (based on^57^). In each condition 14 trails were presented. The side of the hidden reward and the two conditions were balanced and semi-randomized with side and condition not being presented more than twice in a row (and this could not happen at the very beginning of the test trials). At the beginning, the first two trials the reward was always hidden to alternated sides. The 28 trials could be completed at one time, however it was possible to divide the 28 trials to more than one session with a maximum of two optional, approx. 10 min long breaks (laboratory cats N = 33; no break: 15, one break: 9, two breaks: 9 cats; laboratory dogs N = 22; no break: 15, one break: 5, two breaks: 2 dogs). In case of a break, there were two motivation trials before the new test trials. The duration of the pointing tests greatly varied between species and location depending on their performance and the presence or absence of breaks; cat lab: approx. 10–40 min, cat home: approx. 35–50 min, dog lab: approx. 15–30 min. Thus we did not examine the effect of breaks statistically.

The O kneeled at a predetermined point and held the subject by its harness or its body. The E kneeled approx. 2.8–2.9 m away from the subject, facing towards it. She held two identical plastic containers scented with the food in front of her body and placed the piece of food into one of them, shuffled them in her hands twice (to prevent the subject knowing in which container the food was) and then placed them on the floor (one after the other, always in the same order—to the right and left from the subject’s point of view) 2.5 m away from the subject, 2.5 m apart from each other, so that she was approx. 30–40 cm behind the midline between them. The distance between the tip of the index finger and the bowl was about 80 cm in both conditions (DDS and DM) (Fig. 1). We chose to place the bowls 2.5 m apart to have the 80 cm distance from the subject’s point of view and therefore the subjects really needed to extend the pointing finger towards the pointed bowl to succeed in the task, which resembles more to a real life referential communication situation.

The material of the plastic containers ensured that the subjects could not hear the food bits moving in the containers when placed on the ground. Both containers have been used in the motivation trials and hence have been scented by the time of the pointing trials. Further, Polgár et al.^58^ found that even from distances of 1 m dogs were not successful in choosing the baited pot using only olfactory cues. In the case of cats, Pongrácz and Onofer^48^ ran a scent control test for their A-not-B error test (instead of pointing the E put the hands behind the back) and found that from a distance of 1–1.5 m cats’ success rate was at chance level, so they did not use olfactory cues to choose the hidden food bait from two metal cups.

### Procedure

Before the pointing gestures, the E presented an attention-getting cue (making short, clapping sounds with her mouth or hands, calling the subject’s name or tapping on the floor or wall with her nails) to attract the subject’s attention and attempted to establish eye contact with it. The pointing gestures, that immediately followed the attention-getting cue, were presented when the animal was standing still and clearly looked at (facing) the E.

DDS pointing: E was pointing with one extended arm towards one of the containers, while looking at the subject, until the animal chose. The subject was released 1 s after the E made the pointing gesture.

DM pointing: E was pointing with one extended arm towards one of the containers for about 1 s, then pulled her arm back to her chest and kept looking straight at the subject. The subject was released to choose immediately after the pointing signal.

Three types of outcome were coded for each trial: correct, incorrect, or no-choice. If the subject did not leave the starting position in 5 s after the pointing gesture was enacted, the E repeated the attention-getting cue and the pointing one more time.

Correct: The container first approached by the subject within 10 cm was considered as chosen, but only if the subject was going directly towards it.

Incorrect: The subject did not get any food if the empty container was chosen. The E quickly picked up both bowls after the incorrect choice in order to prevent the subject from examining the unchosen one.

No-choice: If the subject did not move in the direction of the containers within another 5 s, then ‘no-choice’ was recorded (no-choice: subject did not approach the bowls or directly approached the E; see^59^). After choosing the baited container the subject was allowed to eat the food and was praised verbally by the O.

After a choice, the dogs could be called back to the starting point, while cats had to be carried back. No-choice trials were not repeated (e.g.,^60^), as repeating such trials would have increased the length of the experiment. If the subject made three no-choice trials in a row during the test, we placed one bowl with food in front of the animal so it could regain motivation (in the laboratory, this happened in the case of 10 cats (once: N = 4; twice: N = 4; 3 times: N = 2) and 2 dogs (only once), and at home in the case of 7 cats (once: N = 4; twice: N = 2; three times: N = 1) from the subjects that made at least 14 choices from the 28 trials).

### Pointing retest

To assess whether within-test experience improved the performance, we retested as many subjects as we could in the laboratory. We retested 15 dogs (mean age ± SD = 51.3 ± 46.7 months; 7 males (4 neutered), 8 females (5 neutered) on average 280 ± 101 days (SD) (range 70–379 days) after the first laboratory test, while cats' laboratory retest had to be cancelled due to the COVID-19 outbreak. The testing procedure was the same as described above, except that only 14 trials (7 from both conditions) were conducted and at least 12 choices were needed to be included in the analysis. Since there was no difference in the success between the first and second half of the 28 trials during the test (see Results), we retested dogs using only 14 trials. The duration of the retests was approx. 5–15 min.

### Data collection and analyses

All sessions were recorded with a camcorder and the videos were later coded using Solomon Coder 19.08.02 (developed by András Péter: http://solomoncoder.com). We coded whether a choice was made (choice vs. no-choice) and the success of choices (correct vs. incorrect). Inter-rater reliability was not assessed, because the subjects’ choices could be determined without ambiguity.

We used R statistical software^61^ version 4.2.1 in RStudio^62^ version ‘2022.7.1.554’ with packages lme4 and effects. Shapiro–Wilk test was used to test for normality.

For the choice analysis, we used a general linear model with binomial distribution to examine the effects of species and pointing condition on the number of choices (here only lab data of both species was considered). Since cats were tested in two locations, a generalized linear mixed model with binomial distribution was used with the trial number, pointing condition, test location and age as fixed factors and cats’ ID as a random factor to examine their effects on the number of cat’s choices (here only cat data of both locations was considered). In this model the interactions of the test location and trial number, and test location and age were included in the beginning, but only the significant interactions remained in the final model.

In the success analysis we had two options. We could either include the no-choice trials as incorrect choices (see^63^), i.e. correct choices would be compared to the combined number of incorrect and no-choice trials. The logic behind this was that if cats were not successful in choosing the correct bowl, then it is irrelevant whether they made a choice or not. Alternatively we could exclude the no choice trials (see^64^), i.e. correct choices would be compared to the number of incorrect choices, which would be an unfair advantage to the cats compared to the dogs. Both options were explored, but we thought that it is only fair to use the success data of those cats that made an effort to choose during the test, so we excluded all cats from the success analysis that made a choice in less than 14 trials (see Supplementary Data). For the success analysis, we used a general linear model with binomial distribution to examine the effects of species and pointing condition on the number of successful choices (here only lab data of both species was considered). Since cats were tested in two locations, a general linear model with binomial distribution was used to examine their effects of trial number, pointing condition, test location and age on the number of cat’s successful choices (here only cat data of both locations was considered and cats successful in both locations were excluded from this analysis).

All success data (cat DDS, cat DM, dog DDS, dog DM) were normally distributed. One sample t-tests were used to compare the number of correct choices to the chance level (50%). The individual success of cats and dogs was compared using Fisher’s exact test.

The Wilcoxon Signed Ranks test was used to compare dogs’ success in the laboratory between the first and second half of the test, and also to compare test–retest success of dogs in the laboratory; we pooled the DDS and DM data for these analyses.

## Results

### Testability

In the laboratory, all dogs made a choice in at least half of the trials (N = 21), while only 15 out of the 33 cats did the same. At home, 14 cats made a choice in at least half of the trials out of the 19 cats. For the summary of the descriptive results see Supplementary Table S1 for cats and Supplementary Table S2 for dogs.

**Figure 2:**
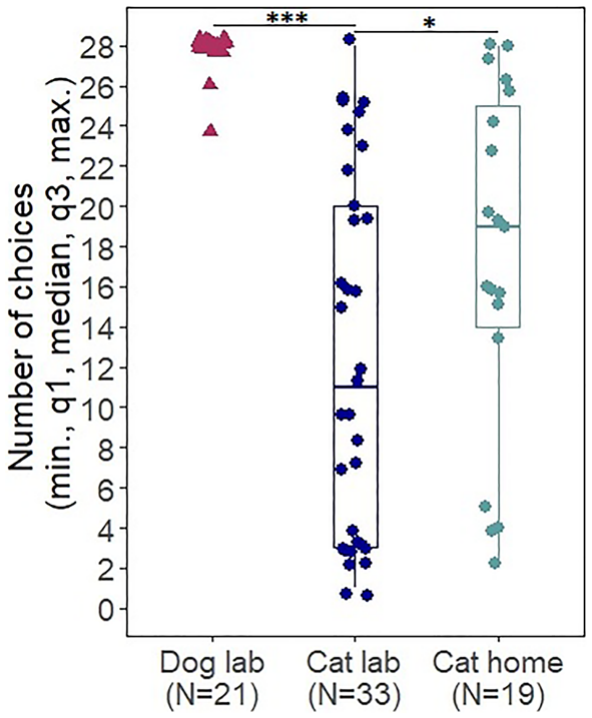
The number of choices (the sum of correct and incorrect) of dogs and cats in the laboratory and the subsample of cats, which were also tested at home (median, quartiles, minimum/maximum excluding outliers). The dots represent the individual data points. All cats are represented in this graph (cats tested: only in the laboratory, only at home and in both locations). * *p < *0.05; *** *p < *0.001.

The number of choices was affected by the species (est = − 3.429, SE = 0.426, z = − 8.05, *p < *0.001), and was not affected by the pointing condition (est = 0.114, SE = 0.22, z = 0.518, *p* = 0.604). Thus dogs chose significantly more than cats in the lab (Fig. 2).

**Figure 3:**
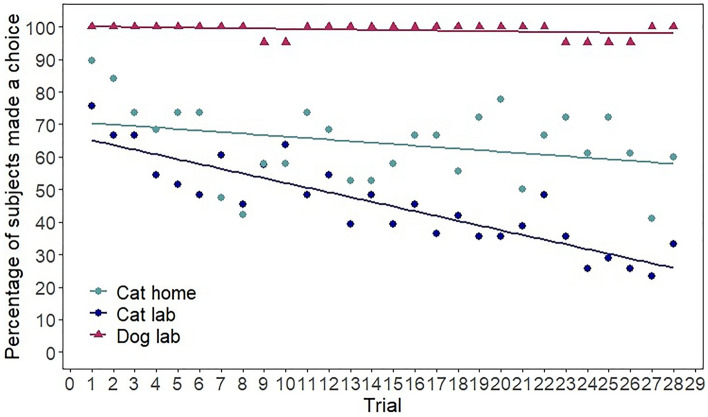
Percent of subjects making a choice in the laboratory and at home in a given trial. Regression lines show the change in the number of choices over the trials. As the number of cats that were presented by a given trial was not exactly the same, here we give the number of cats by trials. Laboratory trials: trials 1–17 (N = 33), trials 18–26 (N = 31), trial 27 (N = 30) and trial 28 (N = 24). Home trials: trials 1–15 (N = 19), trials 16–26 (N = 18), trial 27 (N = 17) and trial 28 (N = 15). The number of dogs in each trial was N = 21.

Cats’ number of choices was affected by test location (est = 1.231, SE = 0.493, z = 2.499, *p* = 0.012) and there was a significant interaction of trial number and test location (est = − 0.093, SE = 0.024, z = − 3.924, *p < *0.001). Thus cats chose less in the laboratory (see Fig. 2) and as the trials progressed their choices declined in the laboratory, but not at home (see Fig. 3). Cats’ number of choices was not affected by the trial number (est = − 0.02, SE = 0.016, z = − 1.267, *p* = 0.205), the pointing condition (est = 0.153, SE = 0.18, z = 0.85, *p* = 0.395), and the age (est = 0.009, SE = 0.007, z = 1.405, *p* = 0.16).

### Success rate

#### Dogs vs cats at the group level in laboratory

**Figure 4:**
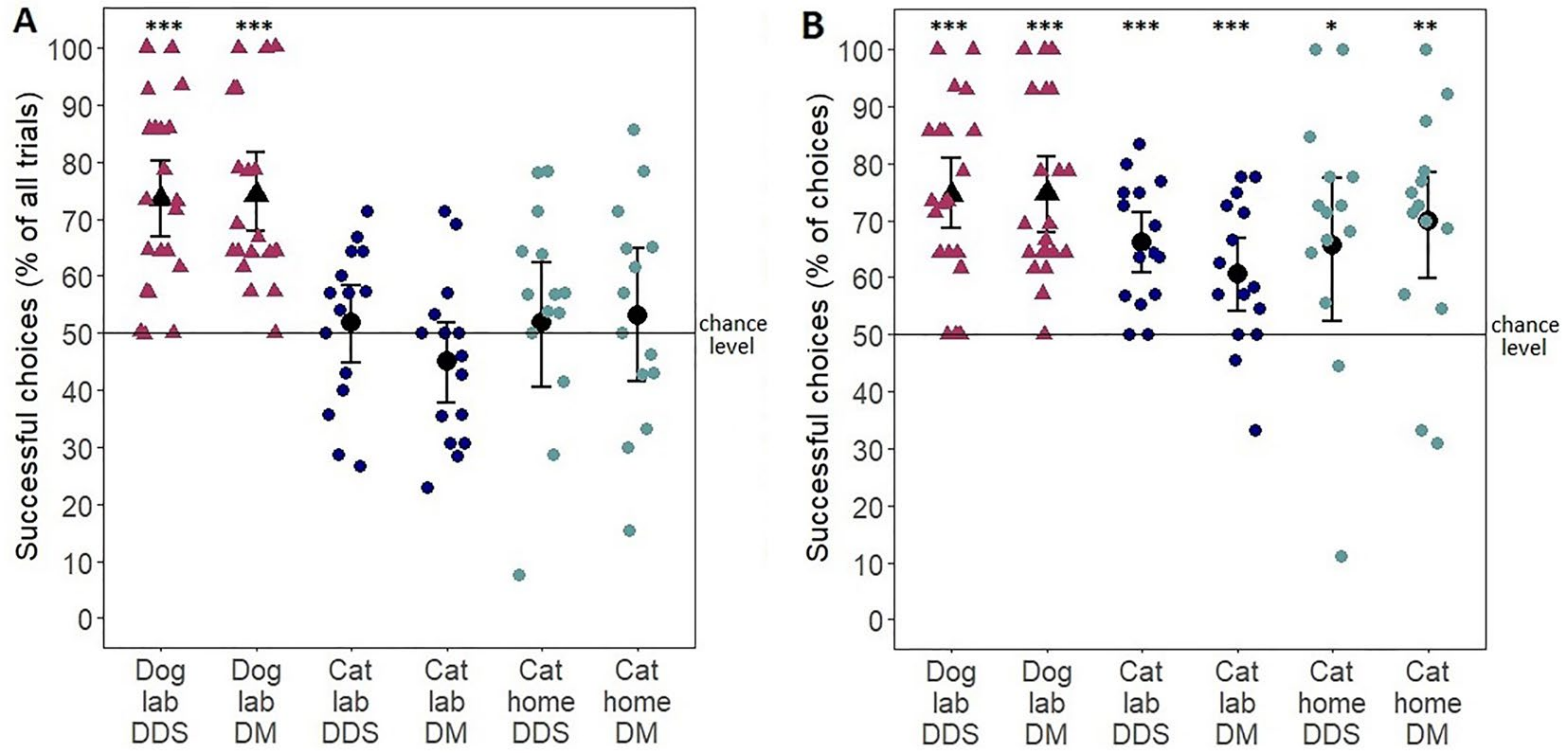
Mean percent of correct choices of the cats (made a choice in at least half of the trials) in the laboratory (N = 15) and at home (N = 14), and the dogs (N = 21) in the laboratory in the two conditions. (**A**) no-choices were considered as incorrect choices, (**B**) no-choices were not considered. Dots represent individual results. (There were only three cats that made a choice in at least half of the trials in both locations.) Difference from chance level: * *p < *0.05; ** *p < *0.01; *** *p < *0.001.

When we considered the no-choices as incorrect choices, the number of successful choices was affected by the species (est = − 1.109, SE = 0.136, z = − 8.17, *p < *0.001), but not by the pointing condition (est = 0.121, SE = 0.136, z = 0.889, *p* = 0.374). Dogs performed significantly above chance level in both conditions, but cats did not (cat DDS: *p* = 0.641; cat DM: *p* = 0.199; dog DDS: *p < *0.001; dog DM: *p < *0.001; see Fig. 4A).

When the no-choices were not considered as incorrect choices, the result was similar to the above. The number of successful choices was affected by the species (est = − 0.519, SE = 0.151, z = − 3.442, *p < *0.001), but not by the pointing condition (est = 0.113, SE = 0.148, z = 0.764, *p* = 0.445). However, in this case both cats and dogs performed significantly above chance level in both conditions (cat DDS: *p < *0.001; cat DM: *p < *0.001; dog DDS: *p < *0.001; dog DM: *p < *0.001; see Fig. 4B).

#### Cats in the laboratory vs at home

The success rate of the 3 cats that made a choice in at least half of the trials in both the laboratory and at home can be found in Fig. 4 and in Supplementary Table S1. These cats in the laboratory had a mean success of 68.04 ± 7.87% (SE) in the DDS and 61.28 ± 14.05% (SE) in the DM pointing, while at home they had a mean success of 82.6 ± 9.11% (SE) in the DDS and 66.67 ± 20.03% (SE) in the DM pointing. Due to the low sample size statistical comparison could not be done.

However, when we excluded these 3 cats from the dataset applying a between-subject design approach, cats’ number of successful choices were affected by age (est = 0.011, SE = 0.003, z = 4.14, *p < *0.001) and trial number (est = − 0.034, SE = 0.01, z = − 3.33, *p < *0.001), but not by pointing condition (est = 0.106, SE = 0.162, z = 0.657, *p* = 0.511) and test location (est = 0.147, SE = 0.176, z = 0.835, *p* = 0.404). Thus older cats had more successful choices and the number of successful choices declined as the trials progressed. Cats did not perform above chance level in any locations and conditions (cat home DDS: *p* = 0.758; cat home DM: *p* = 0.619; cat laboratory DDS: *p* = 0.534; cat laboratory DM: *p* = 0.287; see Fig. 4A).

When the no-choices were not considered as incorrect choices, the number of cats’ successful choices was affected by the age (est = 0.008, SE = 0.003, z = 2.611, *p* = 0.009), but not by the trial number (est = − 0.014, SE = 0.012, z = − 1.194, *p* = 0.233), the pointing condition (est = 0.023, SE = 0.192, z = 0.122, *p* = 0.903) and the test location (est = 0.001, SE = 0.214, z = 0.003, *p* = 0.997). In this case cats performed significantly above chance level in both locations and conditions (cat home DDS: *p* = 0.049; cat home DM: *p* = 0.002; cat laboratory DDS: *p < *0.001; cat laboratory DM: *p* = 0.005; see Fig. 4B).

#### Individual success in the laboratory

Since there was no difference between the results of the pointing conditions, we combined their results for the comparison of the individual success rates of the two species. Eleven dogs (52.4%) performed better than chance level (≥ 20 correct out of the 28 trials), while none of the cats performed better than chance level. Individual success in the lab differed between dogs and cats (Fisher-test, *p < *0.001). When we did not consider no-choices as incorrect choices, 3 cats (7%) performed better than chance level, but the individual success difference remained significant between dogs and cats (Fisher-test, *p < *0.001).

### Retest success

There was no difference in the dogs’ success between the first and second half of the 28 trials during the test (N = 21, z = − 1.537, *p* = 0.124), therefore in the retest only 14 trials were done and compared to the first 14 trials of the test. Dogs’ success (N = 15) in their first test (70.08 ± 4.77%, mean ± SE) vs. the retest (69.05 ± 4.81%, mean ± SE) did not differ (z = − 0.28, *p* = 0.779).

## Discussion

In this study our main aim was to directly compare both the testability and the success of companion dogs and cats to rely on distal human pointing gestures in a two-way choice task in a laboratory environment, and to compare cats’ performance in the laboratory vs. at home. Cats were less testable and their success in relying on distal human pointing gestures was worse compared to dogs at both the group and individual level. Although based on our results it is not possible to precisely disentangle the role of the different ecology, domestication and individual developmental history of the two species, all seem to have played a role and resulted in the significantly worse performance of cats. Based on these differences there are a few probable, non-exclusive direct explanations to our findings for cats’ worse performance: insufficient attention and long-term food motivation; satiation and/or fatigue from many trials; unusual/frustrating human handling in the test situation; and that despite the habituation process, they did not feel comfortable enough to perform a task in the laboratory.

In accordance with our expectations, all dogs proved to be testable, which is in line with the findings of Uccheddu et al.^50^ who reported that all dogs passed their habituation process. For cats, Uccheddu et al.^50^ reported a ~ 40% failure rate in the habituation process to accustom them to the laboratory environment. Although in the present study we only used cats that previously had passed the above habituation process^50^, and only ~ 35% of the enrolled cats (N = 15) made a choice in at least half of the trials in the laboratory. Although the proportion of excluded subjects cannot be directly compared to previous studies due to methodological variability, still ~ 61% (N = 14) of cats made a choice in at least half of the trials at home, which is comparable to published data (see Table 1). Thus cats' testability was better at home, which is probably related to their ecological background that they are comfortable in their home environment.

As cats passed both a familiarization and a motivation pretest, they were certainly motivated by the food, associated the bowl with the food, and also paid attention to the E. We used the individual cats’ favourite treats that could be the most motivating for a given animal, so this way, we even gave cats some advantage over dogs, as dogs uniformly got the same hypoallergenic dog treats. However, it is important to note that dogs may not be just motivated by the food, but also by their natural tendency to cooperate with humans^65^. However, we acknowledge that the cats in our study were probably more sociable than typical cats due to the requirement to interact with the experimenter during the habituation process^50^. Also, Miklósi et al.^20^ found that in a similar test situation, compared to dogs, the latency of looking at the owner was greater in cats and they looked shorter at the owner and also at the experimenter, and produced less gaze alterations. Thus dogs may have had an advantage in their motivation and attention keeping compared to cats, which probably originate from the different domestication background of the two species.

Since dogs are social animals, during their domestication process it was easier to select them for cooperation and attention^2^, hence it is not surprising that dogs made a choice in almost every trial and their number of choices did not decline over the course of the test. In contrast, cats’ propensity to make a choice declined in the laboratory, but not at home. Thus cats’ may not be able to sustain their attention as long as dogs and therefore lose motivation to choose. Moreover, previous studies on cats and dogs typically conducted 20–32 pointing trials (e.g.,^20,21,42,43,47^), which is in accordance with the number of trials in the current study. With such a large number of trials, it is important to consider that subjects, apart from losing their attention, may lose motivation during the test either due to satiation (if they are successful in most of the trials), by fatigue (if they are not successful), or boredom. Based on their different ecological background it is known that cats eat many separate meals of small amounts of food during a day^66^, while dogs usually eat large amounts a few times. Although we tried to avoid satiation (especially in cats), providing our subjects with very small bits of treats, we cannot rule out the possibility that some cats might have not been motivated by the food at the end of the test, which was also raised by other studies^20,42^. Further, cats were reported to need a higher rate of reinforcement compared to dogs^42,43^, as sometimes cats stopped participating after a few unsuccessful trials^42^. Though, it is possible that rather than needing more reinforcement, even motivated cats just prefer not to make an effort to get food^67^.

Maros et al.^68^ reported that horses attempted to attack the E after failing in several trials, which could be a sign of frustration. In other paradigms, frustration has also been shown to affect the behavior of the subjects during the test in the form of unexpected behavior, delayed reaction or reduced performance^69,70^. For example, subjects may lose their motivation to choose and get frustrated when they are not successful in consecutive trials, therefore we tried to prevent this effect by introducing a motivation trial after three no-choice trials and also optional breaks (if the O deemed necessary) to keep the subjects interested. Cats’ hiding behind the bed during the test at home could be best explained by frustration as they could not be motivated by food anymore. Cats’ frustration may have also resulted from the test procedure, as cats had been reported to tolerate manipulation and restraint only for a very short period of time^71^ due to their domestication history of living at human settlements but not being in close contact or physical interaction with humans^25^. Kraus et al.^43^ also mentioned that compared to dogs, cats had lower tolerance for being restrained, and they had to exclude cats due to struggling against handling. Thus, the decline in cats’ choices in the laboratory was most likely due to the fact that they found the procedure (searching for the food and especially being carried back to the starting point after each trial) more frustrating than dogs that were just called or escorted back to the starting point after trials^72^. Importantly, cats tested at home did not show a significant decline in their choices, which contradicts the reinforcement and motivation-based explanations, and rather supports that cats were uneasy in the laboratory. In contrast, the familiar environment may have been reassuring and did not require exploring, and cats were also more accustomed to being handled by the O at home. The stress related explanation was also supported by the results of Mäses and Wascher^47^ who reported no decline in the choices of cats in their familiar environment when they used food distraction instead of restraint to keep shelter cats at the starting point. Besides the direct biases, the testability of dogs could have been enhanced by the presence of their O, providing a secure base/safe haven in a strange novel environment^73,74^, while the lack of such a role of the O may have contributed to the cats feeling uncomfortable in the laboratory^41^. Cats’ more difficult habituation to the unfamiliar environment may originate from two factors. First, from their ecology, because cats’ home range is fixed around a food resource^31^, while wolves and feral dogs may drift their home range to access a food resource even outside their core territory^32^. Second, from their development and experience, because dogs have more opportunities to explore novel environments (e.g., on walks).

Contrary to our expectation, age did not affect cats’ testability. Since the majority of the cats were younger than 5 years of age this result may not be surprising, as young cats are more open and curious. This skewed sample probably originates from our selection criteria that only those cats could be tested that were habituated to the laboratory environment and the E^50^. Thus to make a thorough assessment of the effect of age, future studies should be conducted.

For a thorough interpretation of the success results and their comparison with previous findings, first the potential effect of the different experimental setups should be discussed, as slight differences in the experimental setup may significantly affect the subject’s performance in the given task^75^. In the only previous comparative study^20^, the distal pointing of the kneeling E may have resembled proximal pointing from the subject’s point of view (see Supplementary Information). In other dog studies, the distance between the two bowls was also smaller and/or the E stood during distal pointing^10,21,23,76,77^ so that her hand could be seen more above the pot than in our set up. The smaller distance between the pointing finger and the object increases the chances to use the pointing hand as a discriminative cue. By placing the food bowls far apart, we ensured that subjects that could extrapolate the vector of the pointing finger were more successful.

Since cats’ number of choices was significantly lower than that of dogs, in the success analysis we included only those cats that made a choice in at least half of the trials. Further, there were two potential methods to analyze cats’ success data. First, no-choices were considered as incorrect choices (e.g., see^63^). With this method, at the group level, cats’ success did not differ from chance level in all test locations and pointing conditions. However, Hare et al.^64^ suggested not to consider no choices as incorrect choices since if a cat did not make a choice, it did not have the chance to be successful. In this alternative analysis, at the group level, cats’ success was significantly above chance level in all test locations and pointing conditions, even in a more challenging setup, in which we ensured the 80 cm distance between the index finger and the bowl, compared to previous studies^43^. Thus, based on the results of the two different methods, cats’ reliance on distal human pointing gestures seem to depend on their motivation to make a choice. In contrast, at the group level, dogs’ performance was above chance level in both locations and pointing conditions similarly to Miklósi et al.^20^.

Similarly to Miklósi et al.^20^, there was no difference in the success between the pointing conditions, which we did not expect, as we considered the DM pointing to be more difficult, because the cue is only visible for a second. Further, cats’ success did not differ between the laboratory and at home tests (between-subject design). The habituation process^50^ may have helped in easing the stress in a relatively unfamiliar environment in the testable cats. Further, this result confirms that there was no significant order effect, as cats were always tested a second time at home and their success did not improve. However, it should be noted that the 3 cats that chose in at least half of the trials at both test locations seemed to have a somewhat higher success in the DDS pointing at home than in the laboratory. This question needs further investigation in a within-subject design study with a larger sample size.

At the group level, dogs’ success in the DDS pointing seems somewhat lower than in other studies^20,77^, though their results in DM pointing were comparable to those reported in some studies^10,20,21,23^, while somewhat lower than in others^22,76^. One could argue that in some studies^22,76^ half of the dogs were reported to have previous experience in pointing tasks from earlier experiments, which may have affected their performance. However, studies investigating within-test changes in dogs’ performance found no learning effect in this paradigm^20,21^, and in the current experiment we supplemented previous results by testing the learning effect by repeating the pointing task on a second occasion, and found no improvement in the dogs’ performance. Due to the strictly kept 80 cm pointing distance, the likelihood of using alternative strategies (instead of relying on the relevant cue) increases in such two-way choice tests. Thus, side-bias^21^ (preferring to constantly choose one of the sides irrespective of the experimenter’s pointing cue) or win-stay^48,58^ (choosing the side where the subject was successful in the previous trial) strategies may also have played a role in the slightly poorer success compared to previous studies (e.g., cats^20^, dogs^20,22,76^).

It must be noted that in comparative studies even similar success in a specific test would not in itself indicate generally similar socio-cognitive skills in two species^78^. In the case of the comprehension of human cues, the ability to rely on more complex gestures has only been systematically investigated in dogs (e.g., pointing with leg and knee, various types of cross pointing with arm, elbow and leg^22,23,76,79^). Some studies attempted to test such complex gestures in other species with more or less success (cats^40^, pigs^80,81^, goats^82^), but those did not apply some standard procedure or were not comparative studies testing multiple species at the same location using the same setup (the same is true for studies with typical pointing gestures, such as proximal/distal dynamic-sustained/momentary), thus there is limited data to provide a general explanation about the differences. Importantly, at the individual level when we considered no-choices as incorrect choices, none of the cats performed better than chance level, which cannot be compared to previous findings as these data have not been reported (e.g.,^20^). In contrast, half of the dogs performed better than chance, which is somewhat higher than the 20–40% reported in DM pointing for young dogs (between 2 and 14 months of age) by Gácsi et al.^21^, which could be best explained by the combined analysis of the DDS and DM pointing conditions in the current study.

Our results, both at the group level and the individual level, supported our hypothesis on the difference in the performance of the two domesticated species, which could be based on differences in the selective environments for the development of communicative, cooperative and inhibitory^10^ skills necessary for successful interactions with humans^2^. Considering the rather similar recent environment dog and cat companions live in, this difference cannot be explained by their relevant experiences.

Developmental factors make the interpretation of our results even more complex. In dogs, it is known that success was not affected by age^21,54^ as they could successfully rely on human distal momentary pointing gestures from two months of age, and that their performance did not significantly change in adulthood. However, here older cats were more successful, which is in accordance with our expectations, but is in contrast to previous findings showing a trend of younger cats being more successful^42^, which can be partly explained by the difference in the treatment of no-choice trials. While Pongrácz et al.^42^ repeated no-choice trials, we considered them as incorrect choices. Further, the age range of the cats was skewed towards the young, which could also influence our results. Importantly, Gácsi et al.^83^ found that unlike dog puppies, wolf pups were not successful in this paradigm, but test-naive adult wolves performed similarly to dogs. Thus, intensively socialized wolves develop a tendency to rely on human distant pointing gestures in the human environment, while dogs exhibit epigenetically enhanced sensitivity for salient human communicative cues. Similarly to wolves, the time spent in the human communicative environment may have enhanced cats’ performance. Unravelling the exact nature of the age-dependent differences in cats’ willingness to participate and success to utilize human pointing gestures requires further investigation.

## Conclusion

Nowadays, due to their role as companion animals, cats receive increased attention in the field of animal-human communication research, and comparative studies with cats and other species (e.g. dogs) are important in assessing the suitability of cats for research purposes. Our findings provide direct evidence that the testability of dogs is significantly better in a two-way choice pointing test in a standard laboratory environment than that of cats, even if cats went through prior habituation. Though cats seem to be able to rely on distal human pointing gestures if they are motivated to choose, dogs’ better performance at both the group and the individual levels supports that they are more attuned to human communicative signals than cats. It is important to note that the cats in this and previous studies were heavily pre-selected, therefore their performance did not represent the behavior and abilities typical of the species (see^84^). Thus, compared to dogs, cats appear to be a less ideal model to study some non-verbal human socio-cognitive abilities in a laboratory environment considering (i) their poorer testability, (ii) their poorer performance in this pointing study, and (iii) the findings of previous studies investigating cats’ testability^13,39,49,85^ and some socio-cognitive abilities (e.g., performance in a problem solving task^20^, in a detour task^85^). In a practical sense, animal model species are employed to gain insight into specific human characteristics. When we assert that dogs are better models for socio-cognitive behavior, we are relying on the rationale that due to their domestication history and sociability, dogs’ behaviour, compared to cats’ behaviour is functionally more similar to that of humans at the population level. This does not rule out the existence of a few individual cats that exhibit dog-like performance in certain tests (when compared to the average performance of dogs), or a few dogs that perform significantly worse. So, when we talk about "a less ideal model" in the case of cats, we are referring to quantity rather than quality.

### Supplementary Information

Supplementary Information 1.

Supplementary Information 2.

## Data Availability

All data are available as supplementary information.
