# Supplementary material for: Dogs outperform cats both in their testability and relying on human pointing gestures: a comparative study
Source: Sci Rep. 2023 Oct 19;13:17837. doi: 10.1038/s41598-023-45008-3 (PMC10587310; doi:10.1038/s41598-023-45008-3)
Supplement: Supplementary file 1 — Supplementary Information 1. [file 41598_2023_45008_MOESM1_ESM.pdf]

Dogs outperform cats both in their testability and relying on human pointing gestures: a comparative study

Attila Salamon[1,*], Stefania Uccheddu[2], Melitta Csepregi[1,3,4], Ádám Miklósi[1,3], Márta Gácsi[1,3]

[1]ELKH-ELTE Comparative Ethology Research Group, Budapest, Hungary
[2]MTA-ELTE Comparative Ethology Research Group, Budapest, Hungary
[3]Department of Ethology, Eötvös Loránd University, Budapest, Hungary
[4]Doctoral School of Biology, Institute of Biology, Eötvös Loránd University, Budapest, Hungary
[*]Correspondence: dr.attila.salamon@gmail.com

Supplementary Information

In the Miklósi et al. (2005) study, the experimenter was kneeling to be low enough to be properly visible for the cats, and she displayed the pointing from farther back behind the line of the two bowls in order to keep the 80 cm pointing distance. Also, she was able to place the two bowls simultaneously, as the distance between the two bowls was 1.3-1.6 m. However, this way, from the subject's point of view, the tip of the pointing finger and the bowl do not look 80 cm apart (see Supplementary Fig. S1), and the task resembles a proximal pointing (<40 cm; see Miklósi and Soproni, 2006).

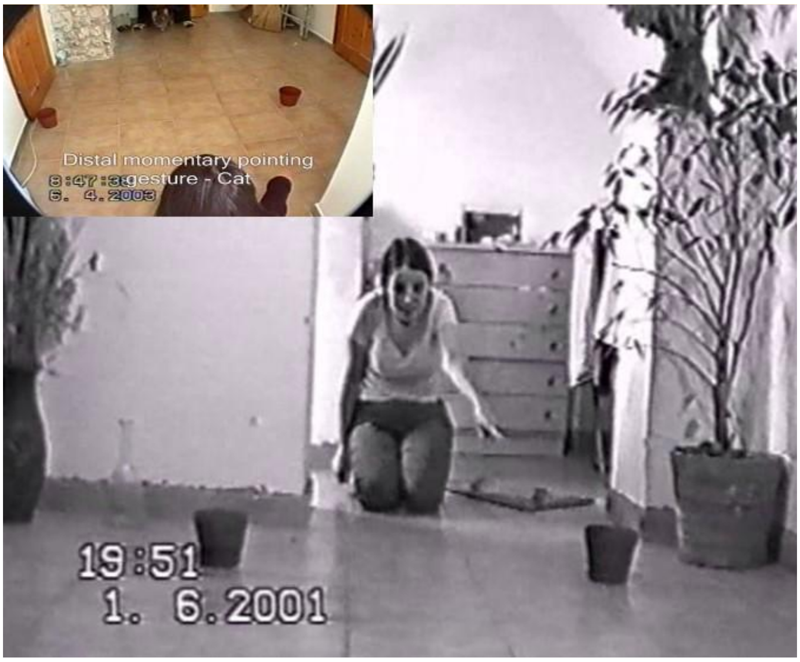

Supplementary Figure S1. ‘Distal’ pointing from the subject’s point of view with an embedded picture showing the experimenter’s point of view (Miklósi et al., 2005). Even though the kneeling experimenter points at the target object from a distance of 70-80 cm, it resembles proximal pointing (<40 cm) from the subject’s point of view. Presented with courtesy of the authors.

One way to keep the 80 cm distance would be to make the E stand up and keep the bowls in the same place, hence have the 80 cm distance almost vertically. In this way the pointing finger may be almost above the pointed bowl (Supplementary Fig. S2), which does not require the extrapolation of the vector of the pointing finger.

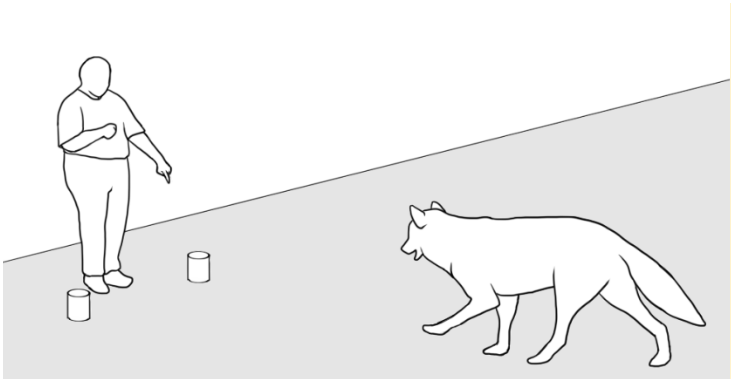

Supplementary Figure S2. Pointing gesture reproduced based on Udell et al. (2008). The pointing finger is almost above the pointed object. This type of pointing does not require much extrapolation of the vector of the pointing finger and therefore the subject may use a simpler strategy to solve the two-way object choice task.

References:

Miklósi, Á., Pongrácz, P., Lakatos, G., Topál, J. & Csányi, V. A comparative study of the use of visual communicative signals in interactions between dogs (*Canis familiaris*) and humans and cats (Felis catus) and humans. *J. Comp. Psychol.* **119,** 179-186 (2005).

Miklósi, Á. & Soproni, K. A comparative analysis of animals' understanding of the human pointing gesture. *Anim. Cogn.* **9,** 81-93 (2006).

Udell, M. A., Dorey, N. R. & Wynne, C. D. Wolves outperform dogs in following human social cues. *Anim. Behav.* **76,** 1767-1773 (2008).

Supplementary Table S1. The test location and results of the enrolled 43 cats. In the laboratory, 10 cats were excluded (7 before the test - failed motivation test or did not come out of the box, 2 during the test - health problem or bit the owner, 1 excluded after the test due to technical problems with the video recording), while 4 cats were excluded at home (2 before the test - failed motivation test, 2 during the test - hid behind the bed). Cats that chose less than 14 times (from the two conditions combined) were not included in the success analyses (laboratory: N=18, at home: N=5). Cats were considered testable when they made a choice at least 14 times out of 28 (laboratory: N=15, at home: N=14) and for these cats the number of correct choices and all choices are shown. Dashes indicate cats that were not tested at home due to COVID-19 outbreak (N=21).

Cat ID	Laboratory		Home	
	DDS (correct / all choices)	DM (correct / all choices)	DDS (correct / all choices)	DM (correct / all choices)
C1	6 / 12	4 / 7	-	-
C2	8 / 10	7 / 9	-	-
C3	7 / 11	8 / 14	-	-
C4	10 / 13	9 / 12	-	-
C5	8 / 14	10 / 14	-	-
C6	4 / 8	5 / 8	-	-
C7	5 / 9	4 / 6	-	-
C8	9 / 12	6 / 12	-	-
C9	6 / 8	4 / 8	-	-
C10	9 / 14	5 / 11	-	-
C11	9 / 13	7 / 12	-	-
C12	8 / 11	6 / 11	-	-
C13	<14 choices combined		failed motivation test	
C14	<14 choices combined		failed motivation test	
C15	<14 choices combined		hid behind the bed	
C16	<14 choices combined		-	-
C17	<14 choices combined		-	-

C18	<14 choices combined		-	-
C19	<14 choices combined		-	-
C20	7 / 11	7 / 9	11 / 14	9 / 13
C21	4 / 7	3 / 9	9 / 13	4 / 13
C22	10 / 12	8 / 11	7 / 7	9 / 9
C23	<14 choices combined		9 / 14	8 / 14
C24	<14 choices combined		8 / 12	8 / 11
C25	<14 choices combined		4 / 9	7 / 10
C26	<14 choices combined		8 / 11	10 / 13
C27	<14 choices combined		10 / 14	11 / 14
C28	<14 choices combined		7 / 9	2 / 6
C29	<14 choices combined		<14 choices combined	
C30	<14 choices combined		<14 choices combined	
C31	<14 choices combined		<14 choices combined	
C32	<14 choices combined		<14 choices combined	
C33	<14 choices combined		<14 choices combined	
C34	failed motivation test		1 / 9	6 / 11
C35	failed motivation test		8 / 11	7 / 8
C36	did not leave the box		11 / 13	12 / 13
C37	failed motivation test		5 / 9	5 / 7

C38	test video not recorded	8 / 8	6 / 8
C39	failed motivation test	hid behind the bed	
C40	health problem	-	-
C41	failed motivation test	-	-
C42	cat bit the owner	-	-
C43	failed motivation test	-	-

Supplementary Table S2. The laboratory test results of the enrolled 21 dogs.

Dog ID	Laboratory	
	DDS (correct / all choices)	DM (correct / all choices)
D1	11 / 15	8 / 13
D2	8 / 11	8 / 13
D3	9 / 14	9 / 14
D4	8 / 13	10 / 15
D5	7 / 14	7 / 14
D6	11 / 14	11 / 14
D7	12 / 14	9 / 14
D8	14 / 14	14 / 14
D9	7 / 14	11 / 14
D10	14 / 14	14 / 14

D11	11 / 15	9 / 13
D12	9 / 14	8 / 14
D13	12 / 14	13 / 14
D14	8 / 13	9 / 13
D15	7 / 14	9 / 14
D16	9 / 14	9 / 14
D17	12 / 14	13 / 14
D18	14 / 15	13 / 13
D19	10 / 14	11 / 14
D20	12 / 14	9 / 14
D21	13 / 14	13 / 14
